# Renal transplantation: Challenges ahead

**DOI:** 10.4103/0970-1591.33716

**Published:** 2007

**Authors:** Nitin S. Kekre

**Affiliations:** Editor, IJU, Department of Urology, Christian Medical College, Vellore - 632 004, Tamil Nadu, India. E-mail: editor@indianjurol.com

The first successful renal transplantation in India was performed on 2^nd^ February 1971 by the team led by Dr. Mohan Rao (Urologist) and Dr. KV Johny (Nephrologist) at Christian Medical College, Vellore. Since then, the program of renal transplantation has come a long way. Today many centers are carrying out organ transplantation. The care of CRF patients has improved due to the easy availability of hemo/peritoneal dialysis. Better immunosuppressive drugs have reduced the complications and improved graft and patient survival. But this treatment is still expensive and a majority of the population cannot afford it. There have been instances of exploitation of the poor for the purpose of organ procurement. Unfortunately, India has earned a dubious name of “Kidney Bazar”. The organ transplantation act which banned unrelated kidney transplantation has not achieved the desired result. There is a need for serious discussion to modify the current organ transplantation act. Cadaver transplantation has not taken off in a big way in this country and the medical community needs to work with the government and social organizations to increase awareness about cadaver transplantation. At the same time, one would have to seriously consider the possibility of improving the live related or unrelated organ transplantation program. Would an Iranian model work for India? There are many challenges which lie ahead in the field of transplantation. Prof. Aneesh Srivastava and his team have addressed some of these issues along with the advances in the field of kidney transplantation. I sincerely thank him for his marvelous effort.

A meeting of experts on prostate cancer was organized recently to resolve controversies and provide definite management guidelines for the treatment of early prostate cancer. At the conclusion of the meeting the experts realized, that they could not provide definitive guidelines, due to lack of good quality evidence. This is not an uncommon situation whenever one looks at the surgical research. We are all aware that the majority of the medical experience never gets published. Whatever remains and gets into print does not always provide good quality evidence. One also needs to seriously wonder about the actual usefulness of published surgical literature. It is for these reasons that surgical research has been sarcastically dubbed the “Comic Opera”. So how does one separate the wheat from the chaff? Dr. Pratap Tharion in the article on “Evaluating systematic reviews in urology” highlights the importance of searching for good quality evidence. It provides information about performing a systematic analysis of the published surgical literature. Most of the articles published on the role of phytotherapy in BPH give us an impression that it is an extremely useful treatment. In the US alone phytotherapy is a million-dollar industry. But a systematic meta-analysis published on the role of phytotherapy concluded that they could not support the usefulness of phytotherapy and suggested the need for well designed prospective randomized control trial to clarify the role of phytotherapy in BPH. Recently Barbagli published a meta-analysis on the buccal mucosa substitution urethroplasty and has commented on the lack of proper evidence to support the superiority of this treatment over others. Dr. Tharion also highlights the value of the Cochrane database and fortunately, free access in available to everyone in india.

In another review, Dr. Shanmugasundaram *et al.* have visited an important clinical problem of testicular microlithiasis and I am sure readers would find this information useful.

From the next issue, I am hopeful of starting a new section which would deal with the history of urology in India and all those who have contributed to the establishment and growth of this specialty. It will not only provide us with historical facts but will also allow the younger generation to acknowledge the contributions of the elders.

